# Screening and bioinformatics analysis of a potential ceRNA network in melatonin-induced cashmere growth in Liaoning cashmere goats

**DOI:** 10.5194/aab-67-97-2024

**Published:** 2024-02-21

**Authors:** Mei Jin, Weiyu Fan, Suhe Lyu, Linlin Cong, Tianwei Xue

**Affiliations:** College of Life Sciences, Liaoning Normal University, Dalian, 116029, China

## Abstract

The purpose of this study was to investigate the effect of melatonin (MT) on the expression patterns of lncRNA, mRNA and miRNA in Liaoning cashmere goat (LCG) skin fibroblasts. A quantity of 200 ng L ${}^{-1}$ MT (MT group) stimulated LCG skin fibroblasts for 48 h, and RNA sequencing was conducted with the control group (Con group) ( n=3 ). The ceRNA network was constructed by bioinformatics analysis of the sequencing data and transmission electron microscopy observation of coated pits and endocytic vesicles. In this study, the results indicated that MT treatment significantly facilitated the proliferation of LCG skin fibroblasts and increased the number of coated pits and vesicles. A total of 775 mRNAs, 57 lncRNAs and 10 miRNAs had differential expression, as indicated by RNA sequencing of skin fibroblasts administrated on the MT group and Con group. The regulatory network of ceRNA was studied, and the results suggested that inositol phosphate metabolism, the cGMP–PKG signaling pathway, endocytosis and other pathways played a certain role in the growth and development of the LCG cashmere. Moreover, the key genes (e.g., *CREB1*, *PIK3C3*, *AGAP3*, *MEF2A*, *ASAP2*, *IRAG1*, *PNISR*, *PIP5K1A*, *SRSF11*, *ZRANB2*, *RBM39* and *CBL*) were regulated by chi-miR-34c-5p, chi-miR-34c-3p and chi-miR-195-5p. The above mRNAs were competitively bound by 15 lncRNAs (e.g., MSTRG.28630.12, MSTRG.28660.14, MSTRG.28099.7). And through dual luciferase and other experiments, it was further confirmed that *PIP5K1A* is the target gene of miR-34c-5p. This finding provides new insights into the molecular mechanism by which melatonin promotes villi growth in cashmere.

## 1 Introduction

The Liaoning cashmere goat (LCG) refers to a precious genetic resource which prohibits gene outflow in China. It has aroused wide attention for its high yield and good quality. The domestic and foreign cashmere market demand for high-grade cashmere has been continuously increasing over the past few years. Melatonin (MT), the main hormone mediating light signal, has been a hotspot in the research field of cashmere growth mechanism. MT alters the cycle of secondary follicle development by regulating the expression of cashmere growth-related genes, while it regulates follicle development by altering the expression pattern of non-coding RNAs. MT and non-coding RNAs take on a great significance in regulating hair follicle development in cashmere goats (Jin et al., 2022), the specific mechanisms of which remain unclear. The LCG skin fibroblasts before and after MT treatment were studied to find out the key genes of MT regulating cashmere growth and further explore the molecular mechanism of MT promoting cashmere growth.

RNA-seq is capable of quickly indicating the expression of most transcripts in tissues or cells under a certain condition while acquiring the information of known or new transcripts (e.g., mRNA, lncRNA and miRNA). The past few years have brought tremendous achievements to RNA-seq analysis in hair follicle morphogenesis, hair follicle development, the regulatory network, the hair follicle cycle, etc. MiRNA refers to a non-coding RNA with a length of 20–22 nt. mRNA post-transcriptional modification is negatively regulated in a wide variety of biological processes. MiRNA–mRNA interaction regulates hair follicle development and the growth cycle. MiR-31-5p facilitates the proliferation of goat hair follicle stem cells and inhibits their apoptosis by targeting the RASA1/MAP3K1 pathway (Feng et al., 2021). Furthermore, chi-miR-30b-5p inhibits the proliferation of dermal papilla cells of LCG by targeting the *CaMKII* δ (Zhang et al., 2020).

LncRNAs are non-coding RNAs over 200 nt in length and lack protein-coding ability (Bridges et al., 2021). Based on their position and orientation relative to protein-coding genes, lncRNAs can be classified into five types: intergenic lncRNAs, intronic lncRNAs, sense lncRNAs, antisense lncRNAs and bidirectional lncRNAs. They are involved in regulating the expression of protein-coding genes and regulating the expression of genes at the transcriptional and post-transcriptional levels (Laurent et al., 2015). LncRNA is capable of interacting with mRNA and miRNA to regulate the expression of downstream target genes. For instance, using whole transcriptome sequencing, it was found that lncRNA-599547 was highly expressed in the dermal papilla of growing LCG. Moreover, lncRNA-599547 adsorbed miR-15b-5p positively regulated *WNT10* expression, such that it is beneficial to induce the dermal papilla cells of Liaoning cashmere goats (Yin et al., 2022). Furthermore, lncRNA XLOC_008679 plays a certain role in the regulation of cashmere properties by targeting *KRT359* (Zheng et al., 2019).

The above results lay a basis for further screening of important candidate genes and molecular markers affecting hair follicle development. However, most of the above studies have focused on the screening, identification and comprehensive analysis of lncRNA and miRNA and the regulation of downstream genes in different growth cycles of cashmere goats, and there has been rare association analysis of the melatonin-regulated lncRNA–miRNA–mRNA ceRNA network. On that basis, this study placed a focus on the differential expression patterns of lncRNA, mRNA and miRNA in skin fibroblasts before and after MT treatment through gene ontology (GO) and Kyoto Encyclopedia of Genes and Genomes (KEGG) pathway analysis. Subsequently, a critical lncRNA–miRNA–mRNA ceRNA network for MT regulation was built based on the interaction of lncRNA–miRNA, lncRNA–mRNA and mRNA–miRNA. This study provides a novel reference for in-depth research on the regulation mechanism of MT promoting cashmere goat's cashmere growth.

## 2 Materials and methods

### 2.1 Cell culture and drug handling

Liaoning cashmere goat skin fibroblasts are cells preserved in this laboratory. The cell culture medium was 20 mL of fetal bovine serum (Gemini, USA), 80 mL of DMEM high-glucose medium (KeyGen Biotech, China) and 1 % penicillin–streptomycin liquid (Solarbio, China). Cells were cultured at 37 ${}^{\circ }$ in a 5 % carbon dioxide incubator (Heal Force, China). Melatonin (Sigma, Japan) was dissolved in DMSO to a concentration of 0.1 g mL ${}^{-1}$ as a stock solution for use. Cells were treated with or without LY294002 (10 µ M) in the presence of melatonin (200 ng L ${}^{-1}$ ) for subsequent detection.

### 2.2 Cell cycle detection by flow cytometry

The cells were digested in the logarithmic growth phase with trypsin, and the cells were resuspended in pre-cooled 70 % ethanol. This was fixed overnight at 4 ${}^{\circ }$ . The fixed cells were collected by centrifuging at 200×g for 10 min, and about 3 µ L of RNase A was added to a final concentration of about 50 µ g mL ${}^{-1}$ , which was digested in a water bath at 37 ${}^{\circ }$ for 30 min. About 50 µ L of PI (Sungene Biotech, China) was added to a final concentration of about 65 µ g mL ${}^{-1}$ , and it was stained in an ice bath for 30 min in the dark. It was filtered through a 300-mesh nylon mesh, and then the cell cycle distribution was analyzed using flow cytometry. The proliferous index (PI) was (S + G2 / M) / (G0 / G1 + S + G2 / M).

### 2.3 Detection of cell apoptosis by flow cytometry

The cells of each group were collected after centrifugation at 150×g for 5 min, and the supernatant was carefully aspirated. The cells were washed twice with PBS, and the cells were collected by centrifugation at 150×g for 5 min. The supernatant was carefully aspirated. The incubation buffer includes 10 mmol L ${}^{-1}$ HEPES/NaOH, pH 7.4, 140 mmol L ${}^{-1}$ NaCl, and 5 mmol L ${}^{-1}$ CaCl ${}_{2}$ . It was washed once, the cells were resuspended with 100 µ L of labeling solution and they were incubated at room temperature in the dark for 15 min. FITC Annexin V (Boehringer Mannheim, Germany) and PI were added to the incubation buffer at a final concentration of 1 µ g mL ${}^{-1}$ . It was centrifuged at 1000 rpm for 5 min to pellet cells and washed once with incubation buffer. Fluorescent (SA-FLOUS) solution was added, and it was incubated at 4 ${}^{\circ }$ C for 20 min. Light was avoided, and it was shaken from time to time. In the flow cytometry analysis, the excitation light wavelength of the flow cytometer was 488 nm, and a passband filter with a wavelength of 515 nm was used to detect FITC fluorescence. Another filter with a wavelength greater than 560 nm was used to detect PI fluorescence.

### 2.4 CCK-8 detects cell proliferation

The Cell Counting Kit-8 (CCK-8; Solarbio, China) was used to detect the proliferation of skin fibroblasts. About 3000 cells per well were seeded in a 96-well plate, and all cells adhered to the wall after 12 h. After the cells were incubated with 10 µ L of CCK-8 solution for 2 h at 37 ${}^{\circ }$ in the dark, cell proliferation was assessed by absorbance at 450 nm using MuLTiSKAN MK3 (Thermo, USA).

### 2.5 Nucleocytoplasmic separation

The instructions of the PARIS™ kit (Invitrogen, USA) were followed. A total of 10 million freshly cultured skin fibroblasts were collected and resuspended in 300 µ L of ice-bathed cell fractionation buffer, and this was incubated on ice for 10 min. It was centrifuged at 500×g for 5 min at 4 ${}^{\circ }$ . The supernatant was the cytoplasmic component, and cytoplasmic RNA was extracted. The precipitate was the nuclear component. A total of 300 µ L cell fractionation buffer was added in the ice bath to the precipitate. It was centrifuged at 500×g for 1 min at 4 ${}^{\circ }$ , and the precipitate was dissolved in cell disruption buffer and vortexed until the lysate became a homogenate. Subsequently, nuclear RNA extraction was performed.

### 2.6 RT-qPCR

Under standard conditions, TRIzol (Vazyme, China) was used to extract cellular RNA, and 1 µ g of RNA was reverse-transcribed into cDNA using the Thermo Scientific™ RevertAid First Strand cDNA Synthesis Kit (Thermo, USA). The expression levels of specific genes were detected by real-time quantitative polymerase chain reaction (RT-PCR) using the TB Green^®^ Premix Ex Taq™ Kit (Takara, Japan), and the data were normalized with β -actin as an internal reference. All data were calculated using the 2 ${}^{-\Delta \Delta \mathrm{Ct}}$ method, and three replicates were detected for each sample. The primer sequences involved in the experiment are shown in Table 1.

**Table 1:** Primer details.

Name	Primer sequence(5 ${}^{\prime }$ – $>3^{\prime }$ )
chi-miR-30f-3p-F	gcctgggaggaggctgtt
chi-miR-215-5p-F	gcttgcgcatgacctatgaattg
R	AGTGCAGGGTCCGAGGTATT
*PIP5K1A*-F	CCGGGCAGCATATCTGAGAG
*PIP5K1A*-R	TGCCGTGGGTCTCTTGATTC
*TNK2*-F	AAGCTGGGAGATGGCTCCTT
*TNK2*-R	CTCAGCACGTCAGGCTTCAG
*HMGXB4*-F	ACCGTGTAACCATTGTGGCTGAC
*HMGXB4*-R	GTCCTTCTCTGGCAACTGCTTCC
β *-actin*-F	CCGTGACATCAAGGAGAAGC
β *-actin*-R	CCGTGTTGGCGTAAAGGT
MSTRG.27945.11-F	CTGTCTGAGCGTCGCTTGCC
MSTRG.27945.11-R	GCGGTCCCCTTCTCCCTTCT
MSTRG.28099.7-F	CCCTCTGGTGACTGTCCTCG
MSTRG.28099.7-R	TCGTCCGGCACGGTTTC
MSTRG.28116.6-F	ACCAGGTGCCCCACGAACG
MSTRG.28116.6-R	GCCCGCGAGAAGGGAGAAGG
MSTRG.28660.14-F	CCTGTCTGAGCGTCGCTTGCC
MSTRG.28660.14-R	CGGTCCCCTTCTCCCTTCTCG
MSTRG.11639.4-F	CAGGTCGGAAACGGAGCA
MSTRG.11639.4-R	AAGAGACGGGGTCTCGCTA
chi-miR-novel-86	RiboBio, China
chi-miR-34c-5p	RiboBio, China

### 2.7 MiRNA inhibitor/mimics transfected cells

The concentration of miRNA inhibitor/mimics (RiboBio, China) was 100 nM. The day before transfection, the cells were digested with trypsin, inserted into a six-well plate (500 000 cells per well) and cultured at 37 ${}^{\circ }$ in 5 % CO ${}_{2}$ . The degree of cell confluence was about 70 %. The medium was removed and replaced with a complete medium without a double antibody. The prepared miRNA inhibitor/mimics was mixed with POLO3000 (Sigma, Japan) transfection reagent, incubated at room temperature for 20 min and then added dropwise to in cells. After 8 h of transfection, it was replaced with a fresh complete medium and cultured in a 37 ${}^{\circ }$ C incubator for 48 h.

### 2.8 Western blotting

Protein lysates of skin fibroblasts were separated by 10 % sodium dodecyl sulfate polyacrylamide gel electrophoresis (SDS-PAGE), transferred to a 0.45 mm transfer membrane (Sigma, USA) and incubated with a specific antibody: anti-p-AKT-Ser473, anti-AKT, anti-Bax (HUABIO, China), anti- β -catenin, anti-Bcl ${}_{2}$ , anti-PIP5K1A, anti- β -actin (Abclonal, China) and anti-GAPDH (Bioworld, China). The dilution rate of the primary antibody was 1:1500 , but the anti-GAPDH was diluted to 1:8000 for immunoblotting. Protein bands were observed with supersignal chemiluminescence substrate (Thermo, USA), and GAPDH or β -actin was used as a control.

### 2.9 Transmission electron microscope (TEM) observation of coated pits and endocytic vesicles

The cultured Liaoning cashmere goat skin fibroblasts were rapidly added with a sufficient amount of precooled 2.5 % glutaraldehyde (Solarbio, China) and fixed for 1 h. They were rinsed with 0.1 M phosphoric acid (Solarbio, China) rinse solution three times, for 15 min each time, and fixed with 1 % osmic acid (Solarbio, China) fixative solution for 1 h, until the sample turned black. They were rinsed with 0.1 M phosphoric acid rinse solution three times, for 15 min each time. In a 4 ${}^{\circ }$ refrigerator, 50 %, 70 % and 90 % ethanol; 90 % ethanol: 90 % acetone ( 1:1 ); and 90 % acetone were used for 20 min each. At room temperature, pure acetone was used three times, each for 20 min. The cells are continuously immersed in the following solution: pure acetone : embedding solution ( 2:1 ) for 4 h at room temperature, pure acetone : embedding solution ( 1:2 ) overnight at room temperature, and pure embedding solution for 3 h at 37 ${}^{\circ }$ C. This was followed by 37 ${}^{\circ }$ C in an oven overnight, a 45 ${}^{\circ }$ C oven for 12 h, and a 60 ${}^{\circ }$ C oven for 48 h. Leica EM UC7 (Germany) sections were 70 nm. After double staining with 2 % uranyl acetate and lead citrate, the sections were observed by an 80 kV TEM (HT7800, Japan).

### 2.10 Functional enrichment analysis

In the present study, use the AnnotationHub package to obtain the annotation information database of *Capra hircus* species (chircus-AH96554). The R package clusterProfiler (Yu et al., 2012) (version 4.2.2) was utilized to perform KEGG pathway enrichment analysis of the gene set. A P value of < 0.05 was regarded as indicative of statistical significance.

### 2.11 Prediction of lncRNA–miRNA interaction

Based on FASTA format sequence files of DElncRNAs and all mature miRNAs, DElncRNA–miRNA interactions were predicted using three algorithms: MiRanda (https://cloud.oebiotech.cn, last access: 12 August 2021), with the threshold set to score > 140 and energy < - 10; lncTAR (http://www.cuilab.cn/lnctar, last access: 12 August 2021), with the threshold set to ndG: - 0.1; and RNAhybrid (https://bibiserv.cebitec.uni-bielefeld.de/rnahybrid, last access: 12 August 2021), with MFE < - 25.0 kcal mol ${}^{-1}$ .

### 2.12 Prediction of miRNA–mRNA interaction

The target genes of up- and down-regulated miRNAs were predicted using three algorithms: miRWalk (http://mirwalk.umm.uni-heidelberg.de/, last access: 16 August 2021); miRDB (https://mirdb.org, last access: 16 August 2021), whereby the binding sites of miRNA and its target were restricted to the 3 ${}^{\prime }$ -UTR region of mRNA, with P≥0.95 and target score > 80; and miRand, with S > = 150, Δ G < = - 30 kcal mol ${}^{-1}$ and demand strict 5 ${}^{\prime }$ seed pairing. CeRNA analysis was performed using the OmicShare tools, a free online platform for data analysis (https://www.omicshare.com/tools, last access: 16 August 2021).

### 2.13 Protein–protein interaction (PPI) network construction, modular analysis and hub gene analysis

A PPI network of 98 genes was analyzed with Search Tool for the Retrieval of Interacting Genes (STRING; http://www.string-db.org/, last access: 18 August 2021) and visualized using Cytoscape 3.9.1. Relationships among 98 genes were analyzed with a network analyzer to characterize the small-world network through calculating the network properties. Molecular complex detection (MCODE) was used to identify key modules within the PPI network using the cutoff criteria (MCODE score > 3) with default parameters (degree cutoff = 2, node score cutoff = 0.2, K core = 2 and max depth = 100). Hub genes in the network were selected using CytoHubba according to connection degree. TBtools (Chen et al., 2020) was used to show the location of DElncRNA on the *Capra hircus* chromosome, and 176 pairs of the down_miRNA–up_mRNA targeting relationship were visualized.

### 2.14 Dual-luciferase reporter gene assays

The 3 ${}^{\prime }$ UTR wild-type sequence of PIP5K1A was obtained based on the goat genome *Capra hircus* (goat) (ARS1.2) in the NCBI database (https://www.ncbi.nlm.nih.gov/genome/?term=Capra+hircus, last access: 26 August 2021). Mutant sites of the complementary sequence of the seed sequence were designed in PIP5K1A 3 ${}^{\prime }$ UTR. A SacI restriction site was introduced at the 5 ${}^{\prime }$ end of the target fragment and a SalI site at the 3 ${}^{\prime }$ end, cloned into the pmirGLO–dual-luciferase reporter. Then, the PIP5K1A-WT + miR-34c-5p mimics, PIP5K1A-MUT + miR-34c-5p mimics, PIP5K1A-WT + NC-mimics, PIP5K1A-MUT + NC mimics, PIP5K1A-WT + miR-34c-5p inhibitor, PIP5K1A-MUT + miR-34c-5p inhibitor, PIP5K1A-WT + NC-inhibitor and PIP5K1A-MUT + NC-inhibitor were co-transfected into 293T cells. After 36 h, 200 µ L dual-luciferase reporter cell lysate was added to each well. The Dual Luciferase Reporter Gene Assay Kit (Beyotime, China) was used to perform the luciferase reporter assay. The ratio of firefly luciferase activity to Renilla luciferase was used as the relative luciferase activity.

### 2.15 Statistical analysis

The data were analyzed by GraphPad Prism version 8.0.2 for Windows. Continuous variables with normal distribution were presented as mean ± standard deviation (SD), and non-normal variables were reported as median (interquartile range). The means of two continuous normally distributed variables were compared by Student's test for independent samples. A value of P<0.05 was considered significant.

## 3 Results

### 3.1 MT treatment promotes the proliferation of skin fibroblasts of Liaoning cashmere goat in vitro

**Figure 1:**
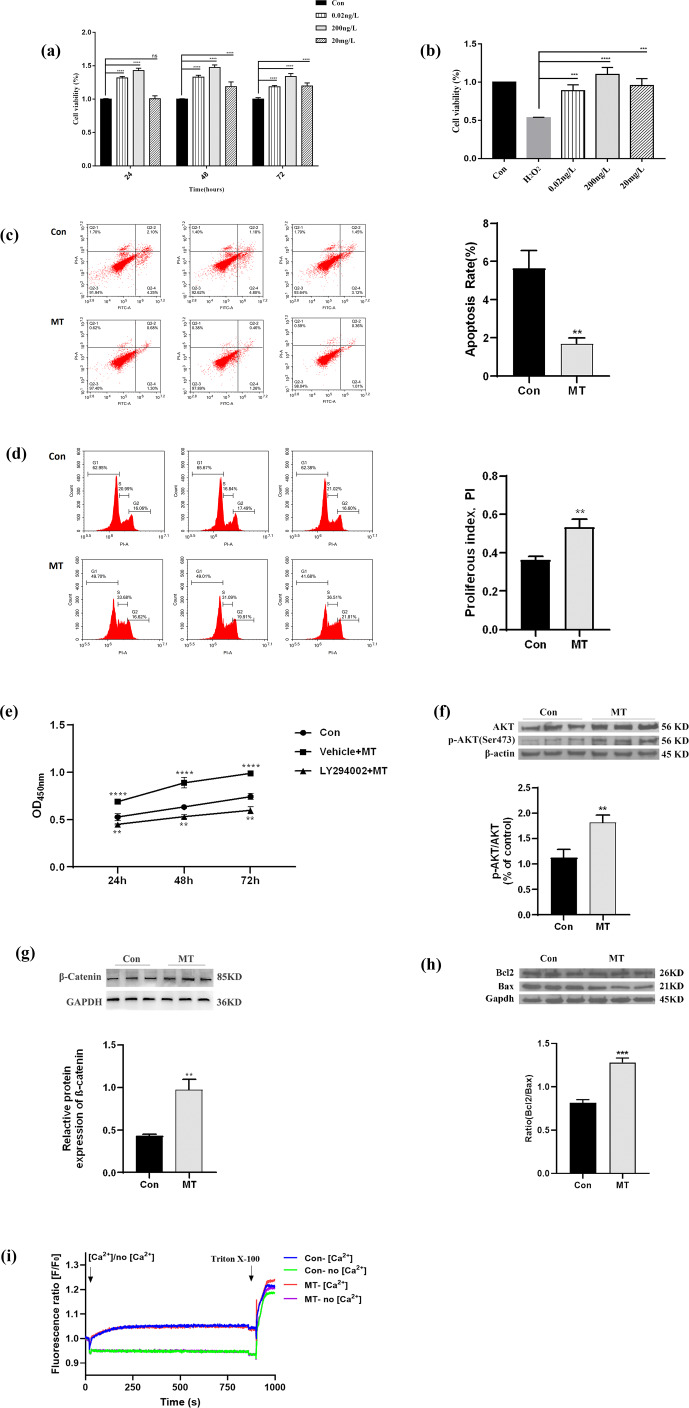
MT treatment promotes the proliferation of skin fibroblasts of Liaoning cashmere goat in vitro. **(a)** CCK-8 assay showed cell viability with different concentrations of MT treated for 24, 48 and 72 h. **(b)** CCK-8 assay showed that MT (0.2 ng L ${}^{-1}$ , 200 ng L ${}^{-1}$ and 20 mg L ${}^{-1})$ treatment for 48 h could prevent the decrease in cell viability of cell from H ${}_{2}$ O ${}_{2}$ (0.65 mM). **(c–d)** Flow cytometry detected cell apoptosis and cell cycle treated with MT (200 ng L ${}^{-1})$ for 48 h, respectively. **(e)** CCK-8 assay showed the effect of PI3K inhibitor (LY294002) on cell proliferation. **(f–h)** WB showed the protein level of p-AKT, AKT, β -catenin and Bcl ${}_{2}$ / Bax in cells treated with MT (200 ng L ${}^{-1}$ ) for 48 h. **(i)** Peak diagram of calcium ion internal flow by fluorescence measurement. The results of CCK-8 and WB were repeated five times, and the results of flow cytometry analysis were repeated at least three times. The result are presented as mean ± SD. Significant results are presented as ${}^{*}$ P<0.05 , ${}^{**}$ P<0.01 , ${}^{***}$ P<0.001 , ${}^{****}$ P<0.0001 in comparison with the control group (Con.); ns is non-significant.

Three MT concentrations (0.2 ng L ${}^{-1}$ , 200 ng L ${}^{-1}$ and 20 mg L ${}^{-1}$ ) and three time points (24, 48, 72 h) were set in the MT effect detection section. 200 ng L ${}^{-1}$ MT treatment significantly increased cell viability (Fig. 1a), whereas 200 ng L ${}^{-1}$ MT treatment significantly ameliorated H ${}_{2}$ O ${}_{2}$ -induced damage (Fig. 1b), as indicated by CCK8 results. Accordingly, 200 ng L ${}^{-1}$ MT treatment for 48 h was chosen for follow-up experiments on skin fibroblasts of LCG. The apoptosis rate in the MT-treated group (1.69 % ± 0.31 %) was significantly lower than that in the control group (5.63 % ± 0.94 %) (Fig. 1c), indicating that MT significantly inhibited the apoptosis ( P<0.01 ). The cell cycle was also examined. The cell proliferation index (PI) of MT-treated group was significantly higher than that of the control group (PI = 0.53 ± 0.04) (Fig. 1d). In addition, the inhibitor of the PI3K-AKT pathway (LY294002) effectively blocked the efficacy of MT (Fig. 1e), as indicated by the results of this study. The expression of PI3K-AKT-pathway-related proteins was analyzed by Western blotting to determine whether MT facilitates cell proliferation in relation to the PI3K-AKT pathway. The results suggested that MT significantly increased the levels of phosphorylated AKT (p-AKT-Ser473), AKT, β -catenin and Bcl ${}_{2}$ / Bax (Fig. 1f–h). Thus, MT can promote cell mitosis, facilitate cell proliferation and inhibit apoptosis through the PI3K-AKT pathway when it is administered with cashmere goat skin fibroblasts in vitro. In order to detect whether melatonin can induce extracellular Ca ${}^{2+}$ influx, frequency-domain spectral shaping (FDSS) detection was performed 48 h after MT treatment of skin fibroblasts. Figure 1i shows that there is no significant change in cytosolic fluorescence intensity (FI) regardless of whether the extracellular medium contains Ca ${}^{2+}$ . This shows that in the presence of extracellular Ca ${}^{2+}$ , MT will not cause intracytoplasmic Ca ${}^{2+}$ overload in skin fibroblasts.

### 3.2 MT treatment altered the expression of lncRNAs, mRNAs and miRNAs

**Figure 2:**
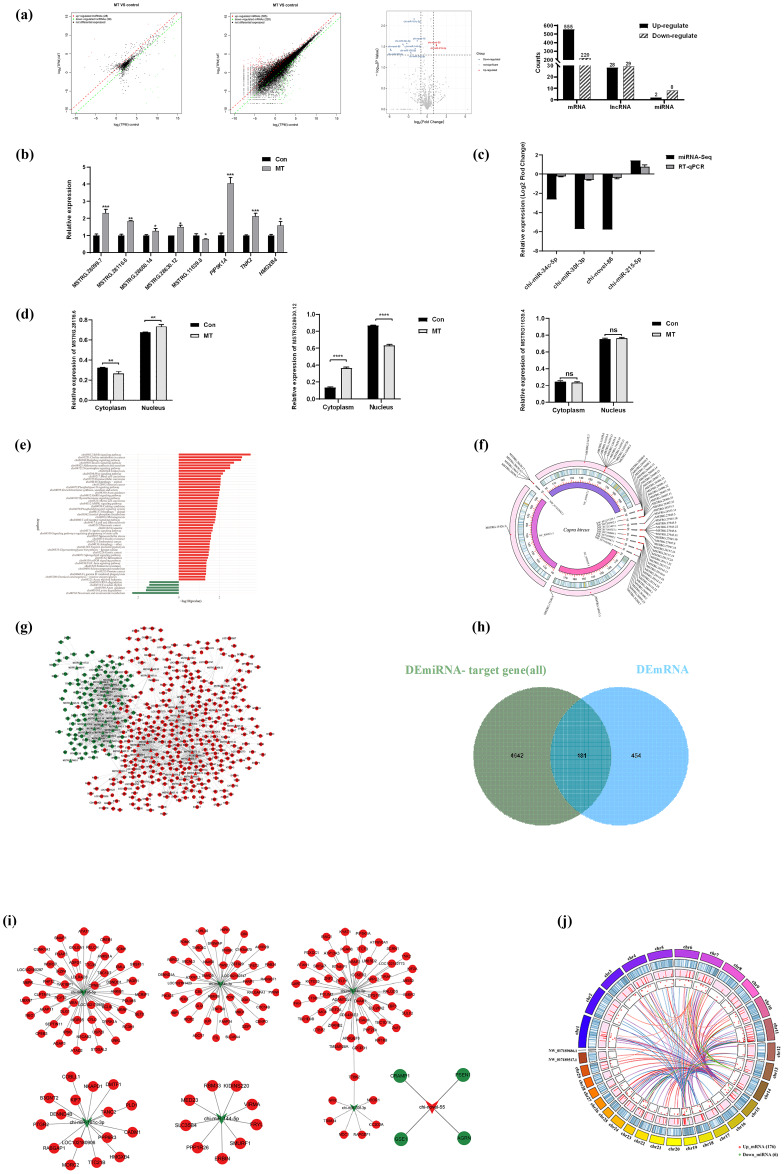
MT treatment altered the expression of lncRNAs, mRNAs and miRNAs. **(a)** Scatter diagram demonstrating DElncRNAs, DEmRNAs and DEmiRNAs between the MT group and control group. **(b–c)** RT-qPCR analysis of the expression changes of five DElncRNAs, three DEmRNAs and four DEmiRNAs before and after MT treatment. **(d)** Subcellular localization of three lncRNAs was detected using a nuclear- cytoplasmic separation assay. **(e)** y axis is the KEGG pathway of differential gene enrichment, and the x axis is log10 ( P value). x>0 indicates the pathway of the up-regulating gene enrichment, and the larger the x axis, the more significant the P value is. x<0 indicates the pathway of down-regulating gene enrichment, and the smaller the x axis is, the more significant the P value is. **(f)** Circos diagram of DElncRNA chromosome distribution. It consists of three different blocks, from outside to inside: chromosome, gene density and DElncRNA position. Red dots represent DElncRNAs. **(g)** DElncRNA–target gene interaction network diagram. Triangles represent lncRNA, nodes represent mRNA, red represents up-regulated expression and green represents down-regulated expression. **(h)** Venn diagram showing overlapping genes both in the DEmiRNA-predicted target genes and DEmRNAs. The overlap areas represent differentially expressed target genes. **(i)** Visualization of miRNA–mRNA network, consisting of 7 miRNA nodes, 180 mRNA nodes and 181 edges. Circular nodes represent mRNAs, and V-shaped nodes represent miRNAs. Red indicates up-regulated expression, green indicates down-regulated expression. **(j)** Circos diagram of down_miRNA–up_mRNA targeting relationship. The chord diagram is composed of four tracks, which respectively represent the distribution of miRNA and mRNA on the chromosome, the expression level of miRNA and mRNA, gene density, and chromosome from the inside to the outside. Each curve in the middle of the donut plot represents the targeting relationship between miRNA RNA and mRNA.

RNA-seq was adopted to detect the expression profiles of lncRNA, mRNA and miRNA in the MT group and the control group, so as to further explore the transcription regulated by MT in the skin fibroblasts of LCG. In this section, the differential expression of mRNA is first analyzed. Combined with the expression level of all mRNAs, the DEmRNAs between the MT group and the control group were screened. As depicted in Fig. 2a, the expression levels of 555 DEmRNAs were significantly increased (up) and 220 DEmRNAs were significantly decreased (down) in the MT group vs. control group. A total of 28 DElncRNAs were up-regulated, 29 DElncRNAs were down-regulated, 2 DEmiRNAs were up-regulated and 8 DEmiRNAs were down-regulated. For RT-qPCR verification, 12 transcripts were randomly selected from DEmRNAs, DElncRNAs and DEmiRNAs in the RNA-seq database. As indicated by the results, MT up-regulated PIP5K1A, TNK2, HMGXB4, MSTRG.28099.7, MSTRG.28116.6, MSTRG.28660.14, MSTRG.28630.12 and miR-215-5p expression and down-regulated MSTRG.11639.8, chi-miR-30f-3p, chi-miR-34c-5p and novel-86 expression (Fig. 2b–c). The expression trends of 12 RNAs were consistent, as indicated by the comparison of qRT-PCR and RNA-Seq data. The reliability of the RNA-seq data was confirmed. Figure 2d shows that after MT treatment, the expression of MSTRG.28116.6 in the cytoplasm decreased, and the expression of MSTRG.28630.12 increased in the cytoplasm. A total of 555 up-regulated mRNAs were primarily concentrated in the signal pathways of cell proliferation, metabolism and growth (e.g., ErbB signaling pathway, Hedgehog pathway, endocytosis, Wnt signaling pathway and phospholipase D signaling pathway), as indicated by the results of KEGG ( P<0.05 ). Besides, 220 down-regulated mRNAs were largely in niacin and nicotinamide metabolism, lysine degradation, axon guidance, RNA degradation, circadian rhythm and other signaling pathways (Fig. 2e). The distribution of 57 DElncRNAs on the chromosome is presented in Fig. 2f, and 45 DElncRNAs were in the in the *Capra hircus* breed San Clemente genome (*Capra hircus* breed San Clemente unplaced genomic scaffold). The DElncRNA–DEmRNA regulatory network (Fig. 2g) was built using Cytoscape, including 491 nodes and 2010 edges, which comprised 1228 pairs of positive and 27 pairs of negative regulatory relationships. The target genes of DEmiRNAs were predicted using RNAhybrid, miRanda and TargetScan software. A total of 181 genes overlapped with DEmRNAs (Fig. 2h). The DEmiRNA–DEmRNA targeting regulatory network was built (Fig. 2i), including 187 nodes and 181 edges, involving 6 down_miRNAs, 176 up_mRNAs, 1 up_miRNA and 4 down_mRNAs, in accordance with the principle that miRNA inhibits the translation of target genes. The result indicated that down-regulated miRNAs constituted a larger network in LCG skin fibroblasts after melatonin treatment, and a Circos diagram was further generated (Fig. 2j). The visualization of 176 pairs of the down_miRNA–up_mRNA targeting relationship provides a novel idea for our further research.

### 3.3 CeRNA network construction

**Figure 3:**
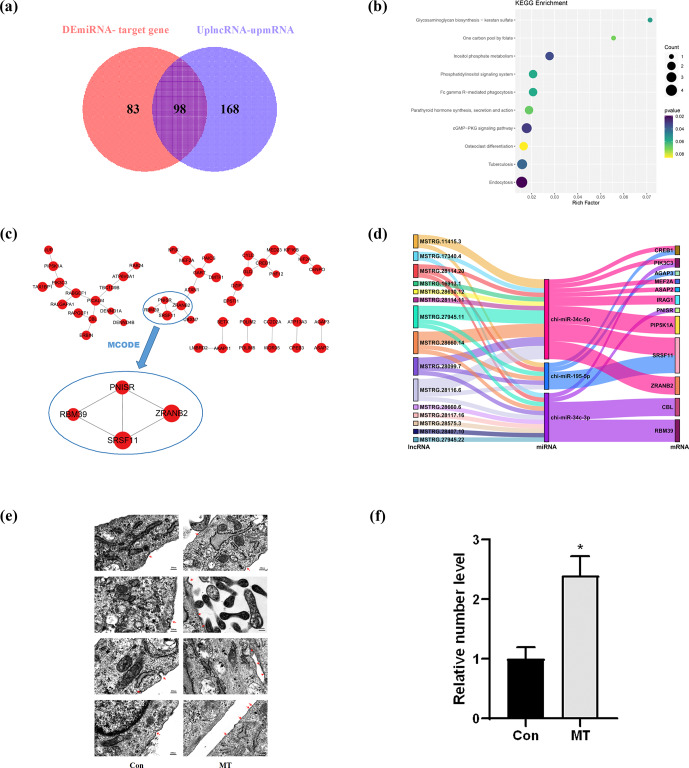
CeRNA network construction. **(a)** Venn diagram identifying mRNAs in the ceRNA network. **(b)** KEGG enrichment analysis of mRNAs involved in the ceRNA network. **(c)** PPI network analysis of mRNAs in the ceRNA network. **(d)** Sankey diagram of the ceRNA network. **(e)** Electron microscopy images of two groups of clathrin-mediated endocytosis (CME). Scale bar is 500 nm. **(f)** The relative number of coated pits and coated vesicle. Significant results are presented as ${}^{*}$ P<0.05 , in comparison with control (Con.) group.

In the ceRNA network, lncRNAs positively regulate mRNAs, and miRNAs negatively regulate mRNAs. Based on this, we screened 98 mRNAs involved in the ceRNA network, namely 98 CEmRNAs (Fig. 3a). As indicated by the results of KEGG functional enrichment analysis, inositol phosphate metabolism, the cGMP–PKG signaling pathway and endocytosis were significantly enriched (Fig. 3b). Moreover, the pathway was significantly correlated with the regulation of intercellular signaling and energy metabolism. There were eight genes involved in the above three pathways. To be specific, endocytosis was the most connected pathway, and PIP5K1A was the most connected gene (Table 2).

The hub gene is a gene that plays a crucial role in biological processes. It often plays a dominant role in regulating other genes in the pathway, and it serves as a vital target and research hotspot. A total of four hub genes were screened, PNISR, ZRANB2, RBM39 and SRSF11 (Fig. 3c), by constructing the PPI network, analyzing the interaction relationship of 98 CEmRNAs and using the MCOD algorithm to analyze the key network modules in the PPI network. There were a total of 12 key genes, including nine genes and four hub genes in the pathway. The focus was placed on the ceRNA regulatory relationship associated with the above 12 key mRNAs from the ceRNA network (Table. S1). The regulatory network of key ceRNAs driven by melatonin was constructed and analyzed. A network of 35 pairs of ceRNAs was built (Fig. 3d), comprising 15 up-regulated lncRNAs, 3 down-regulated miRNAs and 12 up-regulated mRNAs. chi-miR-34c-5p had more lncRNA–miRNA and miRNA–mRNA targeting relationship pairs, which may play an important role in the ceRNA network.

The above findings highlight fundamental features of the melatonin-regulated ceRNA network in endocytosis. The effect of MT on endocytosis was observed in LCG skin fibroblasts using a TEM. As depicted in (Fig. 3e), the images clearly showed the coated ultra-structures of the 200 ng L ${}^{-1}$ MT group and Con group. The coated pits and coated vesicle between the two groups quantity were compared to quantify the above ultra-structures. The number of coated pits and coated vesicle increased 2.4-fold in the MT group ( P=0.01 ) (Fig. 3f), strongly suggesting that MT enhanced endocytosis.

### 3.4 PIP5K1A is a target gene of miR-34c-5p

**Figure 4:**
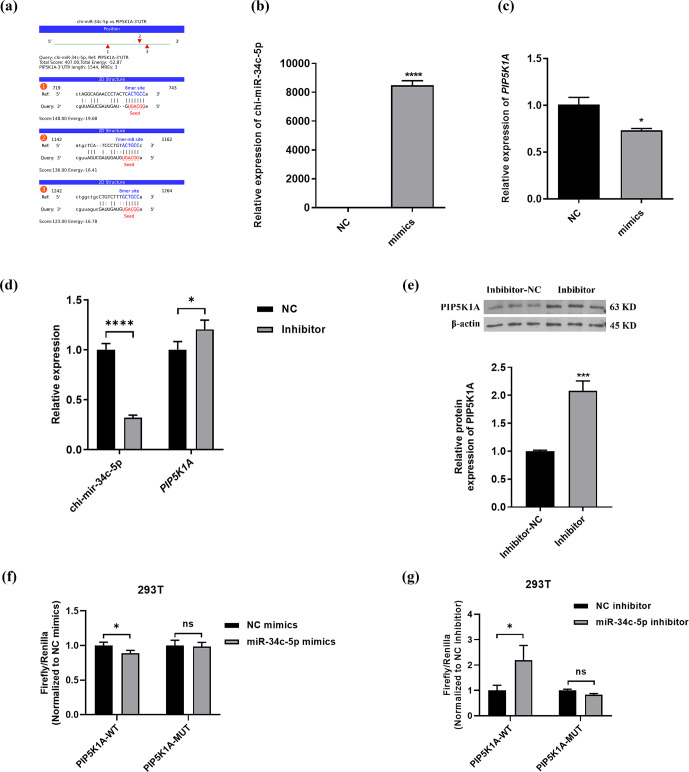
PIP5K1A is a target gene of miR-34c-5p. **(a)** Prediction of binding site between miR-34c-5p and PIP5K1A 3 ${}^{\prime }$ UTR region. **(b–d)** RT-qPCR validation of PIP5K1A and miR-34c-5p expression after transfection with inhibitors and mimics of miR-34c-5p. **(e)** WB showed PIP5K1A expression after transfected with the miR-34c-5p inhibitor. **(f–g)** Detection results of relative activity of the dual-luciferase reporter gene. The result are presented as mean ± SD. Significant results are presented as ${}^{*}$ P<0.05 , ${}^{**}$ P<0.01 , ${}^{***}$ P<0.001 , ${}^{****}$ P<0.0001 in comparison with control group (Con); ns is non-significant.

The binding information of chi-miR-34c-5p and PIP5K1A 3 ${}^{\prime }$ UTR region sequences was predicted through the online miRNA target gene prediction website miRanda (https://cloud.oebiotech.cn/task/detail/array_miranda_ plot/, last access: 28 August 2021). It was found that there are binding sites between the seed sequence 5 ${}^{\prime }$ -GGCAGUG-3 ${}^{\prime }$ of miR-34c-5p and the 736–742, 1152–1161 and 1257–1263 bp regions of the PIP5K1A-3 ${}^{\prime }$ UTR wild-type sequence, as shown in Fig. 4b–c. Moreover, chi-miR-34c-5p mimics significantly down-regulated the expression of PIP5K1A, and the chi-miR-34c-5p inhibitor significantly up-regulated the expression of PIP5K1A gene and protein levels (Fig. 4d–e). It can be seen from Fig. 4f–g that after 48 h of transfection of 293T cells, when the wild-type recombinant plasmid pmirGLO-PIP5K1A-WT group was co-transfected, compared with the inhibitor NC group, the fluorescence activity of the chi-miR-34c-5p group was significantly increased ( P<0.05 ); when the mutant recombinant plasmid pmirGLO-PIP5K1A-MUT group was co-transfected, the difference in fluorescence activity between the chi-miR-34c-5p group and the inhibitor NC group was not significant ( P>0.05 ). When the wild-type recombinant plasmid pmirGLO-PIP5K1A-WT group was co-transfected, compared with the mimics NC group, the fluorescence activity of the chi-miR-34c-5p mimics group was significantly reduced ( P<0.05 ). These findings indicate that miR-34c-5p has a negative regulatory effect on *PIP5K1A*.

**Table 2:** The KEGG analysis results of 98 CEmRNAs.

ID	Description	P value	Gene
chx04144	Endocytosis	$1.8918\times 10^{-2}$	*CBL*/*AGAP3*/*PIP5K1A*/*ASAP2*
chx04022	cGMP–PKG signaling pathway	$2.9961\times 10^{-2}$	*CREB1*/*MEF2A*/*IRAG1*
chx00562	Inositol phosphate metabolism	$3.4100\times 10^{-2}$	*PIK3C3*/*PIP5K1A*

## 4 Discussion

Non-coding RNAs (ncRNAs) constitute a large portion of the transcribed genome and play diverse roles in many cellular processes. NcRNA includes miRNA, circRNA and lncRNA. Many studies have shown that the above ncRNAs participate in competitive regulatory interactions, i.e., ceRNA networks. Accumulating evidence indicates that ceRNA networks are involved in the regulation of hair cycle and hair follicle development. There are numerous factors for cashmere growth. Melatonin is a vital factor for cashmere growth. Melatonin can change the cycle of secondary hair follicle development in cashmere goats by regulating the expression of cashmere growth-related genes. Besides, it can regulate hair follicle development by changing the expression pattern of non-coding RNA. Melatonin and non-coding RNA play a critical role in regulating hair follicle development in cashmere goats, whereas the precise regulation mechanism of lncRNA regulated by MT on hair follicle development-related signaling pathways remains unclear.

Skin fibroblasts before and after MT treatment were employed as transcript sequencing samples and sequenced differentially expressed lncRNA, miRNA and mRNA. Most genes were regulated at the transcriptional level, suggesting that MT is capable of regulating hair follicles by affecting the expression pattern of genetic material development. The above genes may play a certain role in the growth of MT. In our study, 775 differentially expressed mRNAs were found by comparison. Existing research has suggested that the Hedgehog signaling pathway, endocytosis, the Wnt signaling pathway and the TGF- β signaling pathway enriched by KEGG affect the cashmere growth of cashmere goats (Zhang et al., 2022b). Members of the ErbB receptor family serve as the vital regulators of skin development and homeostasis (Hoesl et al., 2019). Zhao et al. (2020) confirmed that IGF-1 and EGF play an active role in hair follicle growth and development. Increased autophagy was detected at the onset of anagen during the natural hair follicle cycle. Chai et al. (2019) found that autophagy is capable of activating quiescent telogen hair follicles and initiating new anagen hair growth. Sadagurski et al. (2006) also demonstrated that phosphatidylinositol 3-kinase and extracellular signal-regulated kinases 1 and 2 play a role in the regulation of skin keratinocyte differentiation. Accordingly, we speculate that the above pathways may be involved in the growth and development of cashmere goats stimulated by MT.

Moreover, 10 differentially expressed miRNAs were identified for the MT group. Existing research has suggested that highly conserved miR-34c-3p/5p, miR-195-5p and miR-144-5p are involved in hair follicle cycle and development (Zhu et al., 2018; Andl et al., 2006; Yuan et al., 2013). Wang et al. have indicated that chi-miR-34c-5p and chi-miR-195-5p were highly expressed in the skin tissue of Shanbei cashmere goats in the telogen stage, and their expression levels were down-regulated in the skin tissue of the growth stage (Wang et al., 2017). Ma et al. (2019) built the XR_310320.3–chi-miR-144-5p–*HOXC8* signaling axis, suggesting that lncRNAs may serve as ceRNAs to adsorb chi-miR-144-5p and indirectly regulate the state of hair follicle stem cells in hair growth. However, the deeper functions of the miRNAs screened in this study should be investigated in depth due to the limited research on the function of miRNAs in the hypothalamus.

There are direct or indirect complex regulatory relationships between cashmere goat lncRNAs and their potential target molecules miRNAs, mRNAs and proteins. lncRNAs exhibit multiple functions (e.g., transcriptional activation, protein-coding gene silencing, and interaction with mRNA or miRNA) to regulate their functions. Existing research has suggested that lncRNA-HOTAIR transcripts may play a certain role in the remodeling of secondary hair follicles during cashmere fiber formation and growth (Jiao et al., 2019).

Over the past few years, lncRNAs have emerged as a previously undiscovered regulator of gene expression that is capable of regulating a variety of cellular processes. Related studies have reported that lncRNAs LNC_000972, LNC_000503 and LNC_000881 may regulate hair follicle cycle through *WNT3A*, *HOXC13* and *MSX235* (Hui et al., 2021). Accumulating evidence has indicated that the effect of lncRNA on mRNA may regulate its function. Existing research has suggested that the relative expression of *ET-1*, *SCF*, *ALP* and *LEF1* in dermal papilla cells can be increased by increasing the expression of lncRNA-000133, which may be correlated with the formation and growth of cashmere fibers (Zheng et al., 2020). Subsequent research has suggested that lncRNA is capable of affecting cashmere growth by interacting with target genes .

In this study, 57 differentially expressed lncRNAs were identified, most of which were located at unique positions in the *Capra hircus* breed San Clemente genome (*Capra hircus* breed San Clemente unplaced genomic scaffold). In-depth analysis of lncRNA functions will be conducive to supplementing and optimizing the lncRNA database of *Capra hircus* species and enriching people's understanding of cashmere growth and development regulation, and it lays a basis for future research. Thus far, the mechanism of MT-mediated ceRNA network regulation of LCG cashmere growth has been rarely reported. Thus, the correlation between lncRNA–miRNA and miRNA–mRNA was predicted using miRanda and TargetScan to further explore the key factors that MT affects the growth regulation of cashmere. The regulatory relationship network of ceRNA network was built based on the correlation between lncRNA–miRNA and mRNA. To be specific, 98 differentially expressed genes were involved. In the functional analysis and PPI network analysis of 98 CEmRNAs, we found that *CREB1, PIK3C3, AGAP3, MEF2A, ASAP2, IRAG1, PNISR, PIP5K1A, PNISR, ZRANB2, RBM39* and *SRSF11* play a key role in the ceRNA network regulated by MT. CREB1 has been confirmed as a vital nuclear transcription factor for eukaryotes. CREB1 facilitates cell proliferation by promoting DNA synthesis in S phase and cell division in G2 phase, and it is also capable of promoting the myogenic differentiation of bovine myoblasts (Feng et al., 2022). And CREB is important for keratinocyte proliferation (Ansari et al., 2008). Adenosine is capable of stimulating the growth of hair follicles by triggering the phosphorylation of CREB (Zhao et al., 2016). PIK3C3/VPS34 (phosphatidylinositol 3-kinase catalytic subunit type 3) forms a complex with BECN1/beclin 1, which takes on a critical significance to the initiation of autophagy important. Besides playing a certain role in autophagosome formation during autophagy, *PIK3C3* is also involved in other cellular processes (e.g., endocytosis, intracellular vesicle trafficking and LC3-associated phagocytosis). The autophagy-related protein PIK3C3/VPS34 controls T cell metabolism and function (Yang et al., 2021). AGAP3 regulates NMDA-receptor-mediated Ras/ERK and Arf6 signaling pathways (Oku and Huganir, 2013). *MEF2A* is a broad distribution of DNA-binding transcription factors. *MEF2A* can inhibit cellular senescence by positively regulating the PI3K-AKT signaling pathway. MiR-615-3p attenuates oxidative stress injury in human cardiomyocytes by targeting PI3K-AKT signaling of *MEF2A * (Zhang et al., 2022a). The CeRNA network in this study by *PNISR* indicated that miR-34c-3p targets *PNISR*. *PNISR* is part of a multiprotein complex in the nucleus, and it plays a certain role in the processing of pre-mRNA to affect cell proliferation and differentiation (Zimowska et al., 2003). *PIP5K1A *acts upstream of the PI3K-AKT pathway to generate PIP2, which is a vital molecule for PI3K to activate AKT (Sarwar et al., 2019). *SRSF11*, a nuclear speck targeting factor, takes on a critical significance to the recruitment of telomerase to telomeres through the interaction with TERC and TRF2 (Lee et al., 2015). *ZRANB2* is an RNA-binding protein, which is combined with *Smad* and inhibits the bone morphogenetic protein (BMP) signaling pathway in HEK293T cells (Jiang and Chen, 2020).

In brief, the transcriptome profiles of LCG skin fibroblasts before and after MT treatment were analyzed; key mRNAs, lncRNAs and miRNAs that might play a certain role in the process of MT-induced cashmere growth were identified; and the MT-mediated ceRNA network consisting of 35 pairs was built. The result indicated that MT up-regulated *PIP5K1A*, *TNK2*, miR-215-5p, MSTRG.28099.7, MSTRG.28116.6, MSTRG.28660.14 and MSTRG.28630.12 expression and down-regulated MSTRG.11639.8, chi-miR-30f-3p, chi-miR-34c-5p and novel-86 expression. The above lncRNAs, miRNAs and genes may play a vital role in the process of MT promoting cashmere growth. This study provides a novel idea for future research on the regulatory mechanism of MT promoting cashmere growth and development.

## 5 Conclusions

The present study was designed to determine the mechanism by which MT promotes the proliferation of LCG skin fibroblasts. A quantity of 200 ng L ${}^{-1}$ MT stimulated cells for 48 h to significantly promote cell proliferation, accompanied by decreased apoptosis, increased endocytosis and activation of the PI3K-AKT pathway. lncRNA as ceRNA participates in MT-mediated skin fibroblast proliferation. Inositol phosphate metabolism, the cGMP–PKG signaling pathway and the endocytosis-related ceRNA network play an important role in the proliferation of skin fibroblasts.

## Supplement

10.5194/aab-67-97-2024-supplement The supplement related to this article is available online at: https://doi.org/10.5194/aab-67-97-2024-supplement.

## Data Availability

The data are available from the corresponding author upon request.

## Code availability

Cytoscape (version 3.9.1) is available at https://cytoscape.org/release_notes_3_9_1.html (Shannon et al., 2003). The R package clusterProfiler (version 4.2.2) is available at https://cran.r-project.org/ (Yu et al., 2012).
