# Supplementary material for: Screening and bioinformatics analysis of a potential ceRNA network in melatonin-induced cashmere growth in Liaoning cashmere goats
Source: Arch Anim Breed. 2024 Feb 21;67(1):97–109. doi: 10.5194/aab-67-97-2024 (PMC12922166; doi:10.5194/aab-67-97-2024)
Supplement: The supplement related to this article is available online at: https://doi.org/10.5194/aab-67-97-2024-supplement. [file aab-67-97-2024-supplement.zip › aab-67-97-2024-supplement-title-page.pdf]

Supplement of Arch. Anim. Breed., 67, 97–109, 2024
https://doi.org/10.5194/aab-67-97-2024-supplement
© Author(s) 2024. CC BY 4.0 License.

Open Access
Archives Animal Breeding

*Supplement of*

# Screening and bioinformatics analysis of a potential ceRNA network in melatonin-induced cashmere growth in Liaoning cashmere goats

**Mei Jin et al.**

*Correspondence to:* Mei Jin (jm6688210@163.com)

- aab-67-97-2024-supplement-title-page.pdf
- Table S1. 12 key mRNAs from the ceRNA network.xlsx

The copyright of individual parts of the supplement might differ from the article licence.
